# The Immunotherapy Landscape in Adrenocortical Cancer

**DOI:** 10.3390/cancers13112660

**Published:** 2021-05-28

**Authors:** Guillaume J. Pegna, Nitin Roper, Rosandra N. Kaplan, Emily Bergsland, Katja Kiseljak-Vassiliades, Mouhammed Amir Habra, Yves Pommier, Jaydira Del Rivero

**Affiliations:** 1 Medical Oncology Service, National Cancer Institute, National Institutes of Health, Bethesda, MD 20892, USA; 2 Developmental Therapeutics Branch, Center for Cancer Research, National Cancer Institute, National Institutes of Health, Bethesda, MD 20892, USA; nitin.roper@nih.gov (N.R.); yves.pommier@nih.gov (Y.P.); jaydira.delrivero@nih.gov (J.D.R.); 3 Pediatric Oncology Branch, National Cancer Institute, National Institutes of Health, Bethesda, MD 20892, USA; rosie.kaplan@nih.gov; 4 Division of Medical Oncology, Department of Medicine, University of California San Francisco, San Francisco, CA 94158, USA; emily.bergsland@ucsf.edu; 5 Division of Endocrinology, Metabolism and Diabetes, University of Colorado School of Medicine, Aurora, CO 80045, USA; katja.kiseljak-vassiliades@cuanschutz.edu; 6 Research Service Veterans Affairs Medical Center, Aurora, CO 80045, USA; 7 Department of Endocrine Neoplasia and Hormonal Disorders, University of Texas MD Anderson Cancer Center, 1515 Holcombe Boulevard, Unit 1461, Houston, TX 77030, USA; mahabra@mdanderson.org

**Keywords:** adrenocortical carcinoma, immunotherapy, immuno-oncology

## Abstract

**Simple Summary:**

Adrenocortical carcinoma is a rare and life-threatening cancer originating from the adrenal glands. Although aggressive surgical interventions may cure this cancer when detected at early stages, treatments for advanced or metastatic disease are limited and often unable to shrink or control the growth of this tumor. Research into new treatments is needed for adrenal cortical carcinoma and other rare cancers. For cancers and other medical conditions, new therapies can be tested through clinical trials, in which patients are treated with new medications or innovative medication combinations. Among these novel and innovative treatments are immunotherapies. These therapies are playing an increasingly important role in the treatment of multiple different cancers. This article focuses on the various immunotherapies that have been, currently are, or will be tested in clinical trials for the treatment of adrenocortical carcinoma.

**Abstract:**

Adrenocortical carcinoma (ACC) is a rare cancer of the adrenal gland that is frequently associated with excess production of adrenal hormones. Although surgical resection may be curative in early-stage disease, few effective therapeutic options exist in the inoperable advanced or metastatic setting. Immunotherapies, inclusive of a broad array of immune-activating and immune-modulating antineoplastic agents, have demonstrated clinical benefit in a wide range of solid and hematologic malignancies. Due to the broad activity across multiple cancer types, there is significant interest in testing these agents in rare tumors, including ACC. Multiple clinical trials evaluating immunotherapies for the treatment of ACC have been conducted, and many more are ongoing or planned. Immunotherapies that have been evaluated in clinical trials for ACC include the immune checkpoint inhibitors pembrolizumab, nivolumab, and avelumab. Other immunotherapies that have been evaluated include the monoclonal antibodies figitumumab and cixutumumab directed against the ACC-expressed insulin-like growth factor 1 (IGF-1) receptor, the recombinant cytotoxin interleukin-13-pseudomonas exotoxin A, and autologous tumor lysate dendritic cell vaccine. These agents have shown modest clinical activity, although nonzero in the case of the immune checkpoint inhibitors. Clinical trials are ongoing to evaluate whether this clinical activity may be augmented through combinations with other immune-acting agents or targeted therapies.

## 1. Introduction

Immuno-oncology is a rapidly expanding field of study focused on the interaction between the human immune system and malignancies, both hematologic and solid tumors, in the context of tumor initiation, proliferation, and progression. Investigation in this field has resulted in the development of immunotherapies directed at unleashing the patient’s immune system to target and destroy cancerous cells. Immunotherapies, therefore, are a class of cancer therapeutics with a distinct antineoplastic approach, in addition to chemotherapy, radiation, and surgical modalities of treatment.

First evaluated in melanoma and renal cell carcinoma, immunotherapies, at times in combination with targeted therapies or chemotherapy, have revolutionized the treatment paradigms of multiple hematologic and solid tumor malignancies in the past decade [1]. Unlike many other therapeutic modalities, certain immunotherapies have proven to be tissue-agnostic, demonstrating activity against tumors without regard to the histologic diagnosis of the cancer or its primary site of origin [2]. Along with the generally favorable side-effect profile of many immunotherapies, their tumor-agnostic nature has made them an attractive approach to clinical research in rare and understudied tumors.

As a broad term inclusive of several immune-targeting strategies aimed at unleashing a patient’s own immune system against malignancies, immunotherapies are frequently subcategorized as either active or passive therapies. This distinction is based upon whether these therapies act through activation of the host immune system (active) or through antineoplastic activity intrinsic to the agent itself (passive) to target tumor cells. Multiple active and passive immunotherapies for adrenocortical carcinoma (ACC) have been and are actively under investigation at this time, and results of these studies are discussed in this review.

## 2. Adrenocortical Carcinoma: Epidemiology, Pathophysiology, and Treatment

Adrenocortical carcinoma (ACC) is a rare malignancy with an estimated incidence of approximately 1–2 per million population per year [3]. The Surveillance, Epidemiology, and End Results (SEER) database provides an estimated incidence of approximately 0.72 per million cases per year in the United States, leading to 0.2% of all cancer deaths nationally [4]. Although ACC can occur at any age, the peak incidence is in the fourth to fifth decades of life [5]. Epidemiologic studies in ACC have suggested a slight female predominance with a female/male ratio ranging from 1.5 to 2.1:1 [6].

ACC frequently presents with signs of excessive adrenal hormone production (Cushing’s syndrome), whereas other presentations include compressive symptoms and pain in approximately 30–40% or incidental diagnosis on imaging studies in 10–15% of cases [7]. In addition to organ failure from metastatic disease, associated Cushing’s syndrome contributes to mortality associated with advanced ACC, as cortisol-secreting tumors have been associated with worse prognosis, limited survival, and higher recurrence rates compared to non-cortisol-producing ACCs [8,9].

### 2.1. Staging and Prognosis

At presentation, ACCs are usually large, measuring on average 10 to 13 cm, with a minority of tumors measuring <6 cm (9–14%), and only 3% presenting as lesions <4 cm [10,11,12,13]. Biochemically or clinically apparent adrenocortical hormone production is evident in up to 45 to 70% of patients, although syndromes of hormone excess are often not readily recognized by physicians, leading to delay in diagnosis and treatments [10,11,14]. Although prognosis varies widely based upon tumor staging (five-year disease-specific survival of 82% vs. 13% in Stage I and Stage IV disease, respectively), most patients will have advanced disease at diagnosis, resulting in an approximately 50% 5-year relative survival rate across stages (Table 1) [15,16].

### 2.2. Current Therapeutic Approaches

For localized primary or recurrent tumors, the treatment of choice is radical surgical resection, offering the best chance for prolonged recurrence-free survival [17]. However, patients with recurrent or metastatic disease are infrequently curable by surgery alone, and relapse is common even in patients without objective and biochemical evidence of residual tumor after initial surgical management [18].

Systemic chemotherapeutic options for ACC have remained limited and difficult to evaluate in this rare disease. Mitotane, an analog of the insecticide dichlorodiphenyltrichloroethane (DTT), has been used for treatment of advanced ACC since the 1960s [19]. It is frequently prescribed in the adjuvant setting, although its activity remains controversial [18,20,21]. Similarly, radiation therapy is frequently used in the adjuvant setting for those patients considered at high risk for local recurrence and in the palliative setting for patients with metastatic or advanced disease [22].

In advanced disease not amenable to surgical management, cytotoxic drugs combined with mitotane are used. Two frequently used regimens include the combination of etoposide, doxorubicin, cisplatin plus mitotane (EDP-M), and streptozotocin plus mitotane (S-M) [23]. These regimens were compared in an international phase-III trial. EDP-M resulted in increased objective response rates (ORRs) and progression-free survival (PFS) compared with S-M; however, no significant difference in overall survival was demonstrated [24]. Multiple targeted therapies have been studied in ACC, including sunitinib, sorafenib, axitinib, linsitinib, and IGF-1 inhibitors; however, all have shown a limited response rate. No targeted therapies have been shown to yield significant therapeutic benefit when studied prospectively. Nevertheless, there are three separate ongoing clinical trials with the aim of studying the safety and efficacy of cabozantinib in ACC [11,25,26,27].

### 2.3. Genomics

While most cases of ACC are sporadic, approximately 10% are thought to occur in the setting of familial cancer syndrome. The most common familial syndromes include Li–Fraumeni syndrome and Lynch syndrome, with rare cases associated with multiple endocrine neoplasia type 1, Beckwith–Wiedemann syndrome, Familial Adenomatous Polyposis, and succinate dehydrogenase (SDHx) mutations [28,29,30,31,32,33,34,35].

The genetics and epigenetic alterations underlying sporadic cases of ACC are not well explored. Previous data and recent ACC global -omics profiling studies reveal frequently detected genetic and epigenetic alterations, including loss of heterozygosity at 17p13, alterations at the 11p15 locus, and mutations in *TP53*, *CTNNB1*, *ZNRF34*, *CDKN2A*, *RB1*, *MEN1*, *PRKAR1A*, *RPL22*, *TERF2*, *CCNE1*, and *NF1* [36,37,38,39,40,41,42,43,44,45]. Decreased expression of MLH1, MSH2, MSH6, and/or PMS2 consistent with high microsatellite instability/mismatch repair protein deficiency (MSI-H/MMR-D) status have also been reported, and ACC is considered to be a Lynch-syndrome-associated malignancy [46].

## 3. Immunotherapeutic Approaches to ACC

As immunotherapies are among the most rapidly developing fields in oncology, there has been a great interest in their potential application in the treatment of ACC. This potential is rendered even more notable when considering the anti-immunogenic nature of many ACCs related to their frequent secretion of immunomodulating steroid hormones, which can limit effective T-cell-mediated adaptive immune response [7]. Results from completed prospective immunotherapy trials in ACC are summarized in Table 2.

### 3.1. Active Immunotherapies

**Figure 1:**
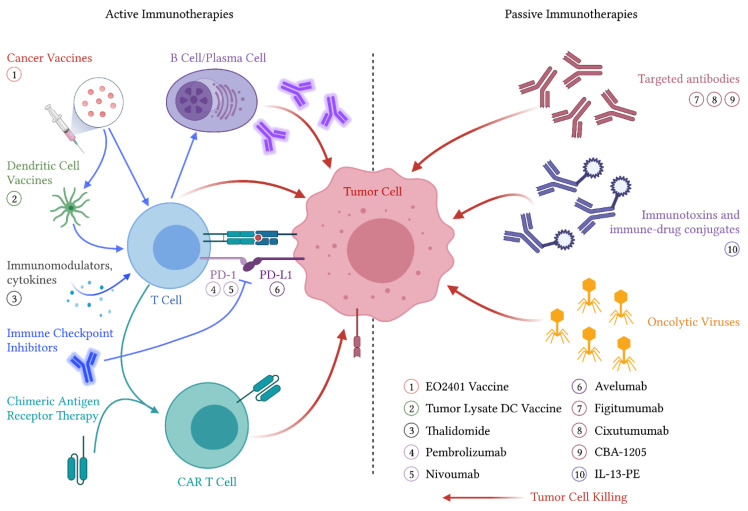
Immunotherapeutic treatment modalities for ACC. Agents that have been evaluated in clinical trials are labeled proximal to their associated mechanism of action. Abbreviations: PD-1—programmed cell death protein 1, PD-L1—programmed death-ligand 1, CAR—chimeric antigen receptor, DC—dendritic cell, IL-13-PE—interleukin-13-pseudomonas exotoxin.

Active immunotherapies cause activation or reactivation of the host immune system against malignant tumor cells. This is believed to occur through either antigen presentation or direct immune cell stimulation, as these therapies are typically understood to require the host’s immune system to exert their effects [47]. This rapidly evolving field includes therapies such as cancer vaccines, immune checkpoint inhibitors, and chimeric antigen receptor T-cell therapies, as well as immunostimulatory cytokines (Figure 1). Cancer vaccines are further subdivided into vaccines thought to prevent the development of cancer, such as human papillomavirus vaccines and therapeutic vaccines that treat existing cancers such as Sipuleucel-T for prostate cancer [48,49]. Several active immunotherapies have been studied in both preclinical and clinical settings in ACC.

#### 3.1.1. Cancer Vaccines

**Figure 2:**
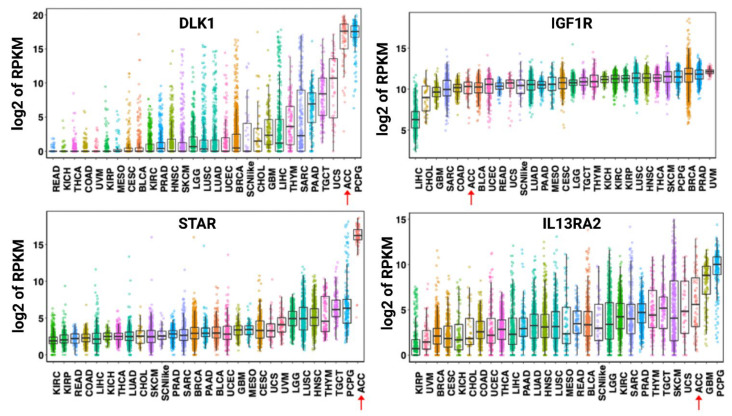
Expression of DLK-1, IGF1R, STAR, and IL13RA2 mRNA across human cancers. Data shown are from the PanCancer RNA-seq data. Each dot refers to an individual tumor. Adrenocortical carcinoma tumors are identified by a red arrow. Abbreviations: RPKM—reads per kilobase million, DLK1—protein delta homolog 1, IGF1R—insulin-like growth factor 1 receptor, STAR—steroidogenic acute regulatory protein, IL13RA2—interleukin-13 receptor subunit alpha-2, LAML—acute myeloid leukemia, ACC—adrenocortical carcinoma, BLCA—bladder, urothelial carcinoma, LGG—brain lower-grade glioma, BRCA—breast invasive carcinoma, CESC—cervical squamous cell carcinoma and endocervical adenocarcinoma, CHOL—cholangiocarcinoma, LCML—chronic myelogenous leukemia, COAD—colon adenocarcinoma, CNTL—controls, ESCA—esophageal carcinoma, GBM—glioblastoma multiforme, HNSC—head and neck squamous cell carcinoma, KICH—kidney chromophobe, KIRC—kidney renal clear cell carcinoma, KIRP—kidney renal papillary cell carcinoma, LIHC—liver hepatocellular carcinoma, LUAD—lung adenocarcinoma, LUSC—lung squamous cell carcinoma, DLBC—lymphoid neoplasm diffuse large B-cell lymphoma, MESO—mesothelioma, MISC—miscellaneous, OV—ovarian serous cystadenocarcinoma, PAAD—pancreatic adenocarcinoma, PCPG—pheochromocytoma and paraganglioma, PRAD—prostate adenocarcinoma, READ—rectum adenocarcinoma, SARC—sarcoma, SKCM—skin cutaneous melanoma, STAD—stomach adenocarcinoma, TGCT—testicular germ cell tumors, THYM—thymoma, THCA—thyroid carcinoma, UCS—uterine carcinosarcoma, UCEC—uterine corpus endometrial carcinoma, UVM—uveal melanoma, SCNlike—small-cell-like tumor. The results shown here are in whole or part based upon data generated by the TCGA Research Network: https://www.cancer.gov/tcga. Accessed on 20 April 2021.

As a malignancy originating from the adrenal cortex, ACC expresses multiple relatively specific antigens that could be evaluated as potential targets for targeted or cell therapies [50,51]. However, expression of many cortex-specific antigens is lost as ACC dedifferentiates [52]. One exception appears to be the steroidogenic acute regulatory protein (StAR), which is maintained across ACC tumors [53]. A high level of StAR mRNA expression has been demonstrated to be highly specific to ACC tumors (Figure 2).

StAR as a therapeutic target has been evaluated in preclinical murine models by Ortman et al. [54]. In this study, repeated injection of plasmid encoding murine StAR (mStAR) followed by injection with a recombinant vaccinia virus expressing mStAR elicited a cytotoxic T-cell response. Antitumor activity was suggested by the decreased tumor development in mice treated with the above regimen compared to controls when given subcutaneous mouse myeloma Sp2-0 cells expressing mStAR [54].

Although StAR-directed vaccines and StAR-based drug delivery antibody–drug conjugates (ADCs) have yet to be tested in clinical trials, another microbiome-antigen (“OncoMimic”)-based vaccine candidate, EO2401 (Enterome SA, Paris, France), is currently being tested in combination with the programmed cell death protein 1 (PD-1) blocking agent nivolumab (Figure 1) in a phase I/II clinical trial (NCT04187404). EO2401 is a cancer peptide vaccine that attempts to utilize homologies between ACC-associated antigens and microbiome-derived peptides to stimulate targeted T-cell mediated tumor killing [55].

#### 3.1.2. Dendritic Cell Therapies

Similar in concept to tumor vaccines, dendritic cell vaccination strategies aim to directly stimulate patient-derived antigen-presenting cells (APCs). Dendritic cells, derived from hematopoietic progenitor cells, act as immune system sentinels, sampling and presenting antigens to T-cells to potentially unmask otherwise undetected microbial and malignant threats [56]. A successful example of this therapeutic strategy is Sipuleucel-T in castration-resistant, metastatic prostate cancer. The use of this therapy, in which autologous peripheral-blood mononuclear cells are stimulated against prostatic acid phosphatase, was shown to improve overall survival in this disease [49].

A difficulty in all vaccine-based strategies for ACC is the lack of identified and validated targetable tumor antigens and, as is the case with StAR, their uncertain immunogenicity [57]. A proposed strategy for circumventing this limitation is to pulse dendritic cells with tumor lysate, therefore exposing these APCs to all known and unknown antigens simultaneously [58]. This strategy was undertaken by Papewalis et al. [59]. Two patients with metastatic ACC had autologous dendritic cells harvested and pulsed with autologous tumor lysate before being reinfused. Although this strategy resulted in increases in T-cell proliferation and induction of cytotoxic T-cells, no impact on tumor growth was observed [59].

#### 3.1.3. Immune Checkpoint Inhibitors

Similar to cancer vaccines, immune checkpoint inhibitors are classified as active immunotherapies acting through direct activation of the host’s immune system. Unlike cancer vaccines, these agents directly inhibit immune suppressive checkpoints. These checkpoints represent critical components of the human immune system, allowing for self-tolerance and the modulation of immune responses to minimize collateral tissue damage. This system of immune regulation has been shown to be co-opted by tumor cells as a mechanism of immune evasion and resistance [60]. Ligand–receptor interactions have been identified as critical components leading to significant and expansive therapeutic developments across multiple solid and hematologic malignancies. Included in these interactions are lymphocyte-bound programmed cell death protein 1 (PD-1) and the tissue- or tumor-bound programmed death-ligand 1 (PD-L1), as well as the T-cell-bound cytotoxic T-lymphocyte-associated protein 4 (CTLA-4) and cluster of differentiation 80 and 86 (CD80, CD86) proteins [61]. By targeting and disrupting these interactions, immune tolerance of tumor cells can be abolished, leading to long-term disease control in certain patients. These advances have been paradigm-shifting in multiple malignancies, including malignant melanoma, renal cell carcinoma, non-small-cell lung cancers, and multiple others [62,63,64,65]. Single-agent and combinatorial immune checkpoint inhibitor therapies with and without chemotherapy or targeted agent therapies are an active area of research across multiple tumor types. Defining and predicting which patients are likely to be responders is warranted as these agents can produce autoimmune side effects and remain costly economically.

#### 3.1.4. Immune Checkpoint Inhibitors in ACC

Two clinical trials using the PD-1 inhibitor pembrolizumab as monotherapy in ACC have been reported. The study by Raj et al. was a phase II clinical trial evaluating pembrolizumab 200 mg every 3 weeks without restriction on prior therapies, with a primary endpoint of ORR [66]. Thirty-nine patients were enrolled in this study, with a median follow-up time of 18.8 months. An ORR of 23% was reported with a disease control rate (DCR) of 52% and a median duration of response that was not reached. For patients in the study, the observed median PFS and OS were 2.1 and 24.9 months, respectively. Six patients in the study were noted to have microsatellite-high and/or mismatch repair deficient status (MSI-H/MMR-D), for which pembrolizumab is now FDA approved as tumor-agnostic therapy. ORR between MSI-H/MMR-D and microsatellite stable (MSS) tumors was similar (33% versus 21%, respectively). Compared to other trials, this study did not report on hormonal status or excess of ACC tumors. Treatment was generally well tolerated, with treatment-related adverse events (TRAEs) grades 3 or 4 seen in only 5 of 39 patients

A second study, reported by Habra et al. [67], as a prespecified cohort of a basket phase II clinical trial, investigated pembrolizumab in patients with rare malignancies. In this trial, patients were required to have progressed with a prior line of therapy, and the primary endpoint was nonprogression at 27 weeks. Sixteen patients were included, and the primary endpoint was evaluable in 14 patients. Nonprogression at 27 weeks was seen in 5 of 14 patients (36%), and 2 partial responses were observed. Treatment-related grade 3 or 4 adverse events were seen in 2 of 16 patients, requiring one patient discontinuing study participation. Thirteen of fourteen patients were MSS, and all were negative for PD-L1 staining [67].

In addition to these two clinical trials, a retrospective analysis by Head et al. reported six patients who received pembrolizumab with concurrent mitotane therapy. In this small analysis of pembrolizumab and mitotane combination therapy, it was noted that two of six patients had a partial response, while the remaining four patients had stable disease (DOR 8–19 months). Two of six patients required discontinuation of pembrolizumab due to the development of grade 3 hepatitis in one patient and grade 3 pneumonitis in the other patient [68].

Similar in mechanism to pembrolizumab, nivolumab (Figure 1) is a PD-1 inhibitor approved for multiple solid tumor malignancies. Nivolumab monotherapy was tested in a phase II trial reported by Carneiro et al. [69]. In this trial, the primary endpoint was ORR. Ten patients with metastatic ACC who were previously treated with or declined first-line metastatic therapies were included and received nivolumab 240 mg IV every two weeks. The best response observed in this trial was 1 of 10 patients with an unconfirmed partial response and 2 of 10 patients with stable disease. As with the previously described studies of pembrolizumab, therapy with nivolumab was generally well tolerated [69].

Avelumab is a monoclonal antibody directed toward PD-L1, the ligand-binding partner of PD-1 expressed on tumor cells (Figure 1). This agent has been FDA approved for use in Merkel cell carcinoma and renal and urothelial cancers [70,71,72]. Avelumab has been evaluated in a phase 1b clinical trial by Le Tourneau et al. [73] in patients with metastatic ACC who had progressed after first-line platinum-based therapy. In this trial, 50 patients were treated with avelumab 10 mg/kg regardless of concurrent mitotane therapy (50% of patients). An ORR of 6% was observed, with partial responses in three patients. The best response of stable disease was observed in 21 patients (42%), and median PFS and OS were 2.6 and 10.6 months, respectively. Among evaluable patients, PD-L1 status was not statistically associated with ORR, with 16.7% of PD-L1+ (*n* = 12) and 3.3% of PD-L1- (*n* = 30) patients experiencing an objective response (*p* = 0.192). TRAEs were generally mild, and 12 patients (24%) had an immune-related TRAE of any grade, with 2 grade 3 including pneumonitis (*n* = 1) and adrenal insufficiency (*n* = 1) [73].

#### 3.1.5. Immune Checkpoint Inhibitors Combined with Targeted Therapies

With immune checkpoint inhibitors playing an increasingly prominent role in cancer treatment, a rapidly expanding field of research is investigating how to overcome lack of response and acquired resistance to these therapies. Targeted agents against the vascular endothelial growth factor (VEGF) pathway have been particularly promising in combination with immune checkpoint inhibitor therapies, including axitinib with pembrolizumab for metastatic RCC and bevacizumab with atezolizumab in unresectable hepatocellular carcinoma (HCC) [74,75].

In ACC, the combination of pembrolizumab with the VEGF-targeted multi-kinase inhibitor lenvatinib was described in a small retrospective case series by Bedrose et al. [76]. In this series, eight heavily pretreated patients (median number of prior lines of systemic therapy = 4) with progressive or metastatic ACC were retrospectively analyzed after receiving the combination of pembrolizumab and lenvatinib. The majority of participants failed prior to immune checkpoint therapy or tyrosine kinase inhibitors. The median PFS in these patients was 5.5 months (95% CI 1.8–not reached). Two (25%) patients had a partial response, one (12.5%) patient had stable disease, and five (62.5%) patients had progressive disease. The combination appeared to be well tolerated with no severe toxicities (grade ≥ 3) reported [76].

Of specific concern to the treatment of functional ACCs with immunotherapies is the possibility that excess production of glucocorticoids by the tumors could cause them to be inherently resistant to active immunotherapies that depend on the host’s immune response. In order to overcome this potential inherent resistance, a phase I trial (NCT04373265) is currently ongoing combining the nonsteroidal antiglucocorticoid relacorilant with the PD-1 inhibitor pembrolizumab [77].

#### 3.1.6. Immune Modulators

A distinct class of active immune therapies are the immunomodulators including thalidomide, lenalidomide, and pomalidomide (Figure 1). These agents are believed to act through phosphorylation of the CD28 receptor component of the B7-CD28 complex, resulting in increased Th1 type cytokine release including interferon gamma and interleukin-2, resulting in T-cell and natural killer T-cell activation and proliferation [78,79]. These agents are now FDA approved for use in multiple conditions, including multiple myeloma and non-Hodgkin’s lymphoma [80,81].

The potential utility of thalidomide treatment in ACC was evaluated by Kroiss et al. in a retrospective cohort study of the European Networks for the Study of Adrenal Tumors registry [82]. In this study, 27 patients with progressive or metastatic ACC who had progressed after receiving mitotane were treated with 50–200 mg thalidomide daily. The best response noted was stable disease in 2 patients, while the remaining 25 patients had progressive disease. The median PFS was 11.2 weeks, with a median OS of 36.4 weeks. Thalidomide was generally well tolerated, with fatigue and gastrointestinal upset being the most commonly observed TRAEs [82].

### 3.2. Passive Immunotherapies

Whereas active immunotherapies exert their antineoplastic activity through the host immune system, passive immunotherapies exert direct and intrinsic therapeutic activity against tumor or other target cells. Examples of passive immunotherapies include targeted antibodies, immunotoxins, immune–drug conjugates (ADCs), and oncolytic viruses (Figure 1) [47].

#### 3.2.1. Targeted Antibodies

Among the oldest and most widely used immunotherapies, targeted antibodies are designed to bind receptors on the tumor cell surface directly. This results in blocking the molecular pathways downstream from the receptors, modulation of signaling pathways, and/or induction of apoptosis through multiple mechanisms, including antibody-dependent cellular cytotoxicity (ADCC) [83,84]. Examples of FDA-approved therapies in this class include rituximab for follicular lymphoma, trastuzumab for Her2+ breast cancer, and cetuximab for metastatic Ras-wild type colorectal cancer [85,86,87].

Figitumumab is a fully humanized IgG2 antibody directed against the IGF-1 receptor (IGF-1R). IGF signaling, mediated through the binding of IGF-1 and IGF-2, has been demonstrated to be mitogenic and an important signaling pathway in ACC. High levels of *IGF-1R* mRNA and protein expression have been detected in ACC tumors (Figure 2) [88]. Figitumumab has been shown to bind and downregulate IGF-1R and block activation by IGF-1 and IGF-2 [89]. This agent was evaluated in a phase I trial by Haluska et al. in 14 patients with metastatic ACC. The maximum tolerated dose was 20 mg/kg, and toxicities were generally mild and included hyperglycemia, nausea, fatigue, and anorexia. The best response to treatment observed in this trial was stable disease seen in 8 of 14 patients [90].

Similar in mechanism to figitumumab, cixutumumab (IMC-A12) is a recombinant human IgG1 directed against IGF-1R. This agent was evaluated in combination with mitotane by Lerario et al. [91] in a phase II trial as first-line therapy for patients with advanced or metastatic ACC. In this study, 20 patients were enrolled to receive cixutumumab 10 mg/kg every two weeks with initial mitotane dosing of 2 g daily. The study was terminated early due to slow accrual and limited efficacy. In this study, the primary endpoint of PFS was 6 weeks (range: 2.66–48), and in 20 evaluable patients, the best ORR was a partial response (PR) in 1 patient and stable disease in 7. Toxicities observed included grade 4 hyperglycemia and hyponatremia and one grade 5 multiorgan failure [91].

As the molecular expression profile of ACC becomes further elucidated (Figure 2), potential future antibody targets continue to be uncovered. These include Delta-like homolog 1 (DLK1)/preadipocyte factor 1 (PREF1), a Notch atypical ligand recently found to be highly expressed in human ACC tumors compared to normal adrenal tissues (Figure 2) [92]. A first-in-human trial of the anti-DLK-1 antibody CBA-1205 has been announced by Tokyo-based Chiome Bioscience [93].

#### 3.2.2. Immunotoxins and Antibody–Drug Conjugates

Similar to targeted antibody therapies, immunotoxins and antibody–drug conjugates (ADCs) are agents designed to target a tumor directly with a targeting domain linked to a toxin or chemotherapeutic agent. These agents represent a highly active area of research and drug development with multiple therapies targeting both hematologic and solid tumor malignancies that have been approved by the FDA in recent years [94,95,96].

One such therapy that has been evaluated in ACC is the recombinant cytotoxin interleukin-13-pseudomonas exotoxin A (IL-13-PE). This agent was designed to target the interleukin-13 receptor alpha-2 (IL13RA2), which has been demonstrated to be significantly overexpressed by ACC tumor cells compared to normal adrenal and nonmalignant tissues (Figure 2) [97,98]. Eight patients were enrolled in this phase I trial of systemic intravenous infusion of IL-13-PE [99]. At the maximum tolerated dose (MTD) of 1 μg/kg, dose-limiting toxicities (DLTs) were thrombocytopenia and renal insufficiency. Further, most patients were noted to develop antidrug antibodies following initial treatment, thus limiting the potential clinical utility of this agent. Yet, of the five patients treated at the MTD and evaluable for response, one had stable disease as best response, while the remainder had progressive disease [99].

**Table 2:** Summary of completed prospective clinical trials of active and passive immunotherapies in ACC.

Identifier	Experimental Arm	Phase	Treatment Line	Primary Endpoint	ORR	PFS	Ref
Active immunotherapies							
Unregistered	DCV: tumor lysate	n/a	2nd	POC	0% (0/2)		[59]
NCT02673333	Pembrolizumab	2	All	ORR	23% (9/39)	2.1 mos	[66]
NCT02720484	Nivolumab	2	2nd	ORR	10% (1/10) *	1.8 mos	[69]
NCT01772004	Avelumab	1b	2nd	Safety, ORR	6% (3/50)	2.6 mos	[73]
Passive immunotherapies							
Unregistered	Figitumumab	1	2nd+	Safety, tolerability	0% (0/14)		[89]
NCT00778817	Cixutumumab + mitotane	2	1st	PFS	5% (1/20)	6 wks	[90]
NCT01832974	Interleukin-13-Psm exotoxin	1	2nd+	Safety, tolerability	0% (0/5)		[98]

Abbreviations: ORR—objective response rate, PFS—progression-free survival, Ref—reference, DCV—dendritic cell vaccine, POC—proof of concept, n/a—nonapplicable, mos—months, NP—nonprogression, wks—weeks, Psm—pseudomonas. * Unconfirmed partial response.

## 4. Future Directions

Although the described trials of immunotherapies in ACC have yet to deliver on the potential promise of these agents, it is an active area of research in this difficult-to-treat disease. Recently, the first in vivo model of humanized ACC patient-derived xenograft (PDX) mouse has been reported [100]. This will allow for further investigation into the effect of immune checkpoint inhibitors in the preclinical setting and may open the doors to further explore the various modes of immunotherapy for ACC in experimental laboratory settings [100]. Current and planned clinical trials evaluating immunotherapy are outlined in Table 3. Active areas of research in this field include combinations of immune checkpoint inhibitors, combination tyrosine kinase and immune checkpoint inhibitors, cancer vaccines, and glucocorticoid receptor antagonists combined with immune checkpoint inhibitor therapies.

## 5. Conclusions

ACC is a rare malignancy with a dismal prognosis. In the metastatic setting, chemotherapy options have limited benefit, and to date, none of the available targeted therapies have thus far demonstrated significant clinical or therapeutic benefit. Effective therapies for this disease are sorely needed. Cancer immunotherapies such as immune checkpoint inhibitors alone or in combination with targeted therapies may have benefits in ACC; however, to date, the clinical utility of these agents in unselected populations of patients with ACC has proven limited. Nonetheless, although few in number, that certain patients have experienced clinical benefit with these therapies suggests that in preselected populations, immunotherapies with or without targeted therapies may reasonably be considered in recurrent advanced ACC. Addressing the limitations of these therapies will likely involve further investigation as to the potential role of supraphysiological doses of circulating corticosteroids limiting the efficacy of these agents. Further studies and predictive surface biomarkers and pharmacodynamic response biomarkers are needed to determine which patient would have the greatest benefit to immunotherapies. Here we described the different immunotherapeutic approaches for ACC and their clinical implications.

## Figures and Tables

**Table 1:** Five-year, disease-specific survival rates for ACC patients by tumor stage.

Stage	Description	Survival (%)
I	Disease < 5 cm, without local invasion, nodal or metastatic spread	82
II	Disease > 5 cm, without local invasion, nodal or metastatic spread	61
III	Tumor with local, lymphatic, vena cava, or renal vein invasion	50
IV	Distantly metastatic disease	13
Tumor staging by the 8th edition of the American Joint Committee on Cancer TNM Staging System and European Network for the Study of Adrenal Tumors		

**Table 3:** Active or planned immunotherapy trials in ACC.

NCT Identifier	Phase	Intervention	Key Inclusion Criteria
NCT04373265	I	Relacorilant with pembrolizumab	Advanced unresectable or metastatic ACC, hormonally active
NCT04187404	I/II	Therapeutic vaccine (EO2401) with or without nivolumab	Advanced unresectable or metastatic ACC
NCT02721732	II	Pembrolizumab	Advanced unresectable or metastatic rare solid tumor malignancies, including ACC
NCT04318730	II	Camrelizumab with Apatinib	Advanced unresectable or metastatic ACC, progressive after first-line therapy
NCT03333616	II	Nivolumab with Ipilimumab	Advanced unresectable or metastatic ACC
NCT02834013	II	Nivolumab with Ipilimumab	Advanced unresectable or metastatic ACC, progressive after first-line therapy

## Data Availability

Not applicable.
